# Effective Treatment of Geniospasm: Case Series and Review of the Literature

**DOI:** 10.5334/tohm.141

**Published:** 2020-08-17

**Authors:** Mariam Hull, Mered Parnes

**Affiliations:** 1 Pediatric Movement Disorders Clinic, Blue Bird Circle Clinic for Pediatric Neurology, Section of Pediatric Neurology and Developmental Neuroscience, Texas Children’s Hospital, Houston, Texas, US

**Keywords:** geniospasm, chin trembling, tongue biting, botulinum toxin, hereditary chin tremor

## Abstract

**Background:**

Geniospasm is a rare genetic disorder characterized by paroxysmal rhythmic or irregular movements of the chin and lower lip due to repetitive contractions of the mentalis muscle. Pathophysiology is poorly understood, and optimal treatment has not been established.

**Methods:**

Geniospasm was characterized in a series of patients after evaluation in our clinics, and a comprehensive review of all cases in the medical literature was performed.

**Results:**

We evaluated four patients (1 female) in four families with geniospasm, aged 4 months to 9 years. Bothersome symptoms were present in one patient, who was treated with regular injections of onabotulinumtoxinA, with complete resolution of symptoms and no adverse effects. 9 patients in the literature have had similar outcomes.

**Conclusions:**

Limited data exist with regard to the effective treatment of geniospasm. Several treatments have been used historically, with variable outcomes. Our results, together with those of prior reported cases, demonstrate benefit of the use of botulinum toxin injections for management of this condition.

## Introduction

Geniospasm is a rare disorder characterized by paroxysms of rhythmic or irregular twitch-like, “quivering,” or “trembling” movements of the chin and lower lip due to involuntary repetitive contractions of the mentalis muscle bilaterally. It was first described in the Italian literature by Massaro in 1894 [1,2] and by Stocks in the English literature in 1922 [3], and since that time has been reported in fewer than 50 families worldwide. Symptoms can be mild and unimpairing, or can include more frequent and irksome chin movements or painful and sometimes bloody nocturnal tongue-biting. The disorder can be inherited in an autosomal dominant pattern, or can occur sporadically [4,5]. The genetic basis is as-yet poorly understood. Given the rarity of geniospasm, the literature to date is limited to small case series and case reports. As such, guidance on prognosis and management strategies can be difficult for providers to gather. We describe here four illustrative cases (1 female) and include a review of all reported cases to date in order to provide a concise review on this under-recognized disorder and provide a summary of the current understanding of geniospasm and treatment strategies.

## Methods

We reviewed the medical records of two patients with geniospasm who presented for evaluation at our tertiary-care pediatric movement disorders clinic between 2015 and 2019. Two additional children were evaluated by video and chart review after being seen in the general child neurology clinic at our institution during this time period. Our review included the history, family history and phenomenology of the geniospasm events of the four affected individuals. Videos of all patients were obtained after signing a consent form approved by the Baylor College of Medicine Institutional Review Board. A systematic review of the medical literature was then performed, and all reported cases of geniospasm were reviewed.

## Illustrative Cases

### Patient 1

The patient is a male with borderline IQ, ADHD and mixed receptive-expressive language disorder who presented to our tertiary care pediatric movement disorders clinic at age 9 for evaluation of episodic chin quivering. Onset began at 3.5 months of age and would occur in bursts of 30 minutes to one hour in length. Triggers included excitement and anxiety but would also happen spontaneously. Initially the movements were intermittent with decreased frequency between ages 2–4 years with subsequent further increase in frequency at 7 years of age occurring in bouts lasting 60 minutes throughout the day (up to 15 hours of chin quivering per 24 hours) with resolution during sleep. These movements made him feel very embarrassed and sad as the movements occurred in front of his peers, which made him feel different and would often cause tearfulness related to wishing the movements would stop. There was no family history of similar movements. Home video revealed rhythmic chin trembling that was consistent with the diagnosis of geniospasm (Video Segment 1). Prior to evaluation in the movement disorder clinic, the family was offered low dose clonazepam, however family deferred. OnabotulinumtoxinA injections were pursued in our clinic and titrated to 30 units to each mentalis every 3 months with complete resolution of symptoms and no adverse effects. No other treatments were tried. Genetic material has been collected for comparative whole exome sequencing.

**Video Segment 1:** Home video illustrating geniospasm in a child.

### Patient 2

The patient is a typically-developing male presenting at 7 months of age with chin quivering occurring multiple times per day lasting between 30 minutes to 4 hours at a time since early infancy. No clear triggers reported. Family history is remarkable only for hypnic jerks in the father but no other members with chin quivering. Home video was provided that was consistent with the diagnosis of geniospasm. The movements subsided by 1 year of age, and as of 3 years of age have not recurred. No treatment was pursued and genetic testing has been deferred by the family.

### Patient 3

**Figure 1:**
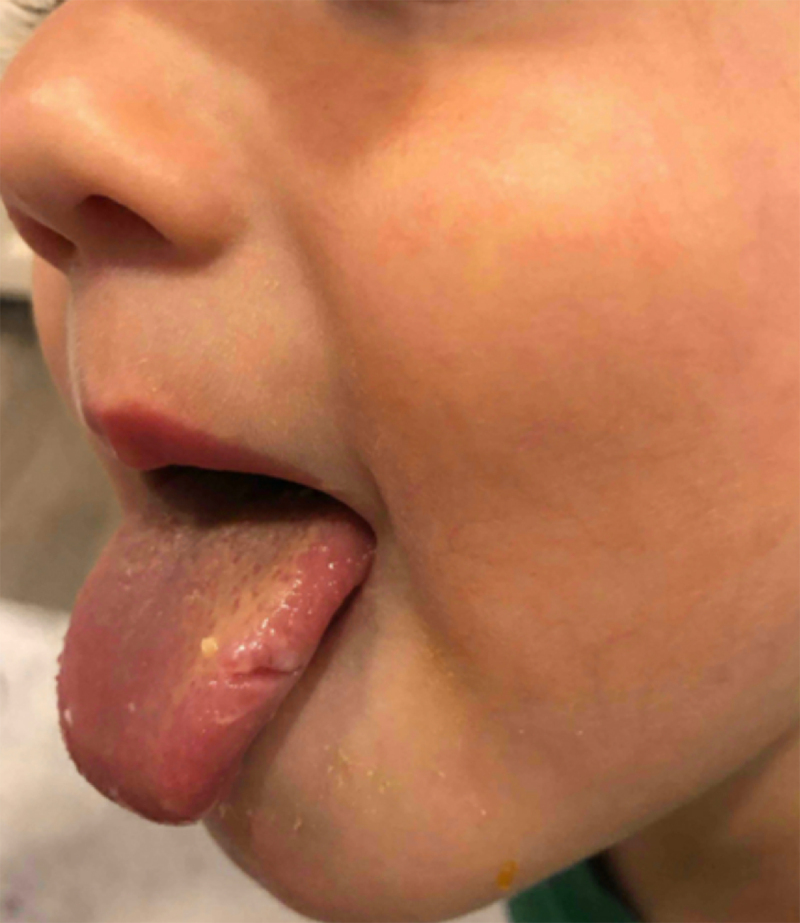
Tongue laceration due to nocturnal tongue biting associated with geniospasm.

The patient is a typically-developing male who presented at 14 months of age for evaluation of nocturnal tongue biting first noted at 11 months of age. He would repeatedly be awakened by oral pain, with blood found on the sheets, up to 25 times per night, leading to repeated ulceration of the tongue (Figure 1). He was also found to have quivering of the chin since his first day of life. It occurs intermittently in bursts of seconds over periods of 30 minutes. No clear triggers noted. His mother and maternal grandfather also report recurrent chin quivering of which persisted into adulthood, as well as recurrent hiccups. The onset of chin quivering in the family members was reported as young adulthood and stress seemed to trigger the movements. None of the family members have received treatment previously. The patient’s mother is planning to receive OnabotulinumtoxinA injections; injections for the patient have been discussed should the movements become bothersome. Genetic material from our patient and the other affected family members has been collected for comparative whole exome sequencing.

### Patient 4

The patient is a typically-developing female who presented at 4 months of age for chin quivering movements first noted on the first day of life which lasted seconds to minutes at a time. These movements occurred while awake and asleep with no clear triggers. She was born full term with an uncomplicated pregnancy and delivery. She has been developing appropriately. There is no family history of similar movements. The movements do not appear bothersome at this time and therefore no treatment discussed. Genetic testing was deferred by the family.

## Results

Among the four patients with geniospasm evaluated at our institution (median age at presentation: 18 months, range: 4 months to 9 years), age of onset for all patients was within the first 6 months of life, and two (50%) had symptoms noted on the first day of life (Table 1). Three of our patients (75%) have no similar family history. One patient (25%) has associated nocturnal tongue-biting. One patient (25%) has cognitive impairments; other patients and their family members have typical development. No triggers have yet been noted in the patients under two years of age; our 9 year-old patient has noted strong emotion makes his events more likely, and the affected relatives of patient three have more events when they are feeling stressed. Affected relatives of patient three are both afflicted with recurrent bouts of hiccups. All patients have nonfocal neurologic exams apart from geniospasm.

**Table 1:** Illustrative cases of geniospasm newly described in this article including pertinent historical details, diagnostics, treatment, and outcome.

PATIENT	AGE OF PRESENTATION	SEX	AGE OF ONSET	TRIGGERS	ASSOCIATED SYMPTOMS	PAST MEDICAL HISTORY	FAMILY HISTORY	DIAGNOSTIC STUDIES	TREATMENT	OUTCOME

1	9 years	M	3.5 months	Strong Emotions	None	ADHD, Borderline IQ, Mixed receptive-expressive language disorder	None	MRI Brain Normal	OnabotulinumtoxinA – 30 U to each mentalis	Complete resolution No adverse effects
2	7 months	M	Early infancy	None	None	Healthy	None	None	Spontaneous remission by 1 year	
3	14 months	M	First day of life	None	Recurrent Nocturnal tongue biting	Healthy	Mother and maternal grandfather with similar symptoms and recurrent hiccups	EEG normal	None	
4		F	Young adulthood	Stress	None	Recurrent hiccups	Mother of patient 3	None	Plan for OnabotulinumtoxinA injections	
5		M	Young adulthood	Stress	None	Recurrent hiccups	Maternal Grandfather of patient 3	None	None	
6	4 months	F	First day of life	None	None	Healthy	None	None	None	

To date, a total of 41 affected patients from 46 families have been clearly described in the English literature (Table 2). Of these, two had no family history of the disorder [4,5]. Age of onset ranged from shortly after birth to late childhood. Of these patients, 26 (63.4%) noted stress or heightened emotion made events more likely. Three (0.07%) were known to have associated tongue-biting. One patient noted improvement with alcohol. Not all cases reported were severe enough to warrant treatment. Of the patients that did receive treatment, 6 were given anticonvulsants. Results ranged from no improvement to some degree of decrease in frequency and duration of the attacks. Six patients were treated with sedatives, also with partial amelioration of symptoms. One patient was treated with a beta blocker, with similar outcome [6]. Botulinum toxin has been used in 9 patients in addition to our own. In all cases, there has been complete or near-complete resolution of symptoms. Injections were well-tolerated apart from one report of asymmetric smile which may have been due to addition of mylohyoid muscle injections [7]. Of note, in the 3 patients with geniospasm and associated tongue-biting, the nocturnal biting seemed to respond well to anticonvulsants or benzodiazepines without clear improvement in the geniospasm [6,8,9].

**Table 2:** Geniospasm cases described in the literature including pertinent historical details, diagnostics, treatment, and outcome.

YEAR OF PUBLICATION	AUTHOR	AGE AT PRESENTATION	SEX	AGE OF ONSET	TRIGGERS	ASSOCIATED SYMPTOMS	PAST MEDICAL HISTORY	FAMILY HISTORY	DIAGNOSTIC STUDIES	TREATMENT	OUTCOMES

1923	Stocks, P [3]	18 years	M	–	Stress	None	Healthy	Two siblings, cousins, and niece with similar symptoms	None	None	

1957	Grossman, BJ [22]	3 years	M	Infancy	Strong emotions	None	Healthy	Father, paternal uncle, paternal grandfather with similar symptoms Father, paternal uncle, and paternal grandmother with otosclerosis	None	None	

1958	Wadlington, WB [25]	40 years	F	Early childhood	Strong emotions	None	Healthy	Father and two sisters with same symptoms and two sons^A,B^ with similar symptoms	EEG normal	Phenytoin 100 mg BID Hydroxyzine 30 mg BID	Some degree of Improvement Some degree of improvement No adverse effects
		9 years	M	8 weeks	None	None	Healthy	Son^A^	None	Phenytoin 10 mg/kg/day Hydroxyzine 20 mg BID	Some degree of improvement Some degree of improvement No adverse effects
		2 years	M	4 months	During sleep, Strong emotions	None	Healthy	Son^B^	None	Phenytoin 10 mg/kg/day Hydroxyzine 10 mg BID	Some degree of improvement Some degree of improvement No adverse effects

1968	Laurance et. al [34]	5 years	M	Infancy	Strong emotions	Trembling impaired speech	Healthy	Maternal grandmother and maternal aunt with similar symptoms	EEG normal Needle EMG – rhythmic discharges of polyphasic complexes at 10 per second	None	
		6 years	M	1 month	None	None	Bifid left kidney, strabismus	Sister, mother, maternal grandmother with similar symptoms	None	None	

1971	Johnson et. al [8]	13 months	M	Infancy	None	Tongue biting	Sleep myoclonus	Twin brother^C^, older brother, father, paternal grandfather, and paternal uncle with similar symptoms Paternal aunt with seizures	Electrolytes, Urine organic acids, Skull films, and EEG normal	Valium	No improvement
		21 months	M	Infancy	None	None	Sleep myoclonus	Twin brother^C^	None	None	

1984	Fahn, S. [35]	30 years	M	Early childhood	None	None	Healthy	Son^D^ with similar symptoms	None	None	
		8 months	M	Infancy	None	None	Healthy	Son^D^	None	None	

1992	Danek, A [16]	13 years	M	Infancy	Stress	None	Somnambulism	Five other family members with similar chin movements One family member with Charcot-Marie-Tooth	Needle EMG – rhythmic polymorphic discharges in the mentalis	None	
		28 years	F	Early childhood	Stress, waking in the morning	None	Migraines	Son^E^ and 10 other family members with similar symptoms	Needle EMG – rhythmic polymorphic discharges in the mentalis	None	
		4 months	M	2 weeks	Before and during breastfeeding	None	Healthy	Son^E^	Needle EMG – rhythmic polymorphic discharges in the mentalis	None	

1992	Gordon et. al [26]	28 years	M	2 weeks	Strong emotions	None	Healthy	Brother^F^, father*, and several paternal uncles with similar symptoms	None	5 units botulinum toxin (Oculinum, Allergan) to each mentalis muscle q2–3 months	Complete resolution of symptoms for 2–3 months following each injection No adverse effects
		8 years	M	Infancy	None	None	Healthy	Brother^F^	None	5 units botulinum toxin (Oculinum, Allergan) to each mentalis muscle q2–3 months	Complete resolution of symptoms for 2–3 months following each injection No adverse effects

1996	Soland et. al [14]	31 years	M	4 years	During sleep, Strong emotions	Trembling impaired speech, drinking, and sleep	Action tremor	16 family members with similar symptoms	CBC, peripheral smear, serum copper and ceruloplasmin normal EMG – during quivering showed motor units of normal morphology firing asynchronously	Variety of medications (unspecified) Botulinum toxin injection (Dysport 60 units) into mentalis on each side	No improvement Complete resolution of symptoms within one week of injections No adverse effects
		38 years	F	Early childhood	Stress, strong emotions	None	Healthy	Sister with nocturnal episodes and tongue biting, 11 other family members with similar chin movements	None	Self resolved by late twenties	

1997	Destee et. al [31]	35 years	M	Infancy	Stress	None	Healthy	Daughter^G^, Brother, mother^H^, nephew^I^, and five cousins with similar symptoms	EEG normal Surface EMG – Sometimes bursts discharged in rhythmically but most often discharge frequency was irregular	None	
		4 years	F	Infancy	During sleep	None	Healthy	Daughter^G^	None	None	
		62 years	F	Infancy	Stress	None	Healthy	Mother^H^	None	Self resolved with time	Occasionally felt shivering of the chin when stressed that was not visible
		11 years	M	Infancy	None	Trembling impaired speech	Healthy	Nephew^I^	None	None	

1998	Bakar et. al [7]	28 years	M	Birth	Strong emotions	None	Healthy	Mother and maternal grandmother with similar symptoms	None	Sedatives and anticonvulsants Botulinum toxin(Botox) injections (25 units) in each mentalis and mylohyoid q4–5 months	Unsatisfactory results Complete resolution of symptoms within two days of injections and lasting 5 months Adverse effects – abnormal appearance of mouth with corners depressing lower lip and center of lower lip elevated which lasted 30–45 days and resolved. Subsequent injection volumes reduced equal dose. No further adverse effects

1999	Diaz et. al [12]	63 years	F	Early childhood	Stress, gazing at flying objects	None	Healthy	28 family members with similar symptoms	Blood count, serum and urine copper, ceruloplasmin normal Surface EMG over mentalis – synchrony of motor unit firing without evidence of denervation Needle EMG – bursts of motor units of normal morphology firing pseudo-rhythmically throughout the muscle at 7–8 Hz	None	

2002	Grimes et. al [32]	15 years	M	Infancy	Fatigue, stress	None	Healthy	Numerous other family members with similar symptoms	Evaluated for changes on the chromosome 9q13-q21 locus through sequencing analysis-Negative	2.5 to 5 units botulinum toxin type A to each mentalis muscle q3–4 months	Complete resolution of symptoms No adverse effects

2006	Devetag et. al [36]	16 years	M	Infancy	Anxiety, stress, tapping the chin	None	Healthy	Brother, grandmother, cousin, paternal aunt with similar symptoms	EEG, Median and trigeminal SEPs normal EMG – arrhythmic spontaneous activity from the mentalis muscle increased after tapping the muscle and disappeared during sleep	Clonazepam Botulinum toxin (Botox, Allergan) 5 units to each mentalis muscle q3–4 months	No improvement Complete resolution of symptoms No adverse effects

2006	Goraya et. al [9]	13 months	M	Infancy	During Sleep	Tongue biting during sleep	Healthy	Father with similar symptoms	EEG normal	Carbamazepine 100 mg BID Clonazepam 0.5 mg BID	No improvement Mild improvement

2007	Erer, S and Jankovic, J [24]	74 years	M	Early childhood	Stress	None	Parkinson’s disease	Two younger brothers with similar symptoms	None	Clonazepam 2 mg BID Bromocriptine 2.5 mg TID Carbidopa/levodopa 25/100 TID	No improvement No improvement No improvement

2007	Papapetropolous, S and Singer, C [4].	15 years	F	Infancy	Feeding, Strong emotions Temper-ature changes	Impaired eating and drinking	Healthy	No family history of abnormal movements	CT/MRI brain, EEG normal	25 units botulinum toxin type A to each mentalis muscle q 9 months	95% improvement in symptoms No adverse effects

2008	Kharraz et. al [10]	70 years	M	Early childhood	Strong emotions, physical stress	None	Healthy	Two daughters^J,K^ with similar symptoms	EMG/NCS – no evidence of myopathic or neuropathic changes. Bilateral synchronous activity exclusively restricted to mentalis. Normal nerve conduction velocities to the chin.	Decreased in frequency with age	
		44 years	F	Early childhood	Strong emotions, physical stress	None	Healthy	Daughter^J^	EMG/NCS as above	None	
		43 years	F	Early childhood	Strong emotions, physical stress	None	Healthy	Daughter^K^	EMG/NCS as above Sleep study – chin trembling during sleep phase 2	None	

2009	Aggarwal et. al [20]	42 years	M	Childhood	None	None	Healthy	Six family members with similar chin movements	EMG/NCS – spontaneous arrhythmic discharges of normal motor units in both mentalis muscle, no peripheral facial nerve hyperexcitability/denervation, presence of bilateral facial nuclear hyperexcitability demonstrated by spread of facial reflex response	Medications (not specified) Left lower peripheral facial nerve surgery 30 units botulinum toxin (Botox, Allergan) to each mentalis q8-10 months)	No improvement No improvement Complete resolution of symptoms No adverse effects

2014	Mahmoudi, M and Kothare, SV [5]	17 years	M	12 years	Sleep	Tongue biting	Healthy	No family history of abnormal movements	CT/MRI brain normal Sleep study captured periods of tremor of chin and lower lip during sleep	Clonazepam 0.5 mg at bedtime	No improvement No adverse effects

2014	Macerollo, A et. al [37]	68 years	M	Early childhood	Strong emotions, concentration	None	Healthy	Daughter^L^ with similar symptoms	None	None	
		37 years	F	Early childhood	Strong emotions, concentration	None	Healthy	Daughter^L^	None	None	
		32 years	F	Early childhood	Strong emotions, concentration	None	Healthy	Several family members with similar symptoms	None	None	

2015	Ehm et. al [13]	40 years	F	Early childhood	Strong emotions	None	Healthy	Six family members with similar symptoms	None	Clonazepam 0.5 mg TID Carbamazepine 100 mg TID	Modest improvement Modest improvement No adverse effects
2015	Jain et. al [33]	5 years	F	Early infancy	None	None	Healthy	Father with similar symptoms	EEG normal Neuroimaging normal	None	

2016	Akiyama et. al [6]	9 years	F	1 week	None	None	Healthy	Mother^M^ with similar symptoms	Electrolytes and thyroid studies normal CT/MRI normal EEG normal EMG – repetitive bursts of muscle activity that decreased during stage 1 sleep and disappeared during stage 2 sleep	Arotinolol (peripherally acting beta blocker with weak alpha blockade) 2.5 mg titrated to 7.5 mg BID	Significant reduction with 2–3 days of symptom free days per week
		36 years	F	Early childhood	Stress	Impaired sleep	Healthy	Mother^M^	None	None	Noted improvement with alcohol

2020	This article	9 years	M	3.5 months	Strong emotions	None	ADHD, borderline IQ, mixed receptive-expressive language disorder	None	MRI brain normal	OnabotulinumtoxinA – 30 U to each mentalis	Complete resolution, no adverse effects
		7 months	M	Early infancy	None	None	Healthy	None	None	Spontaneous remission by 1 year	
		14 months	M	First day of life	None	Nocturnal tongue biting	Healthy	Mother^N^ and maternal grandfather^O^ with similar symptoms and recurrent hiccups	EEG normal	None	
			F	Young adulthood	Stress	None	Recurrent hiccups	Mother^N^	None	Plan for OnabotulinumtoxinA injections	
			M	Young adulthood	Stress	None	Recurrent hiccups	Maternal Grandfather^O^	None	None	
		4 months	F	First day of life	None	None	Healthy	None	None	None	

* Father was also injected with 5 units botulinum toxin (Oculinum, Allergan) to each mentalis muscle interdose interval 2–3 months with complete resolution of symptoms and no adverse effects.

## Discussion

Geniospasm is a paroxysmal movement disorder of the mentalis muscle [10]. It has also been called familial trembling of the chin, hereditary quivering of the chin, hereditary chin trembling, and hereditary essential chin myoclonus [11]. Classically, the chin movements can be precipitated by stress or strong emotions, but can also occur spontaneously [12]. Improvement has been reported with alcohol consumption in at least one case [13]. Onset is in infancy or early childhood [1]. Events may become less frequent with age [14].

The presence of the mentalis muscle allows the lower lip to remain vertical to cover the lower incisors and prevent drooling. Contraction of the mentalis elevates the lower lip and chin, generating a pouty, thoughtful, or resolute facial expression, depending on concurrent actions of other facial muscles [15]. The mentalis is active during speech, pursing of the lips, smiling, whistling, kissing, and mastication [16]. Similar phenomenology to geniospasm can be seen as a prelude to crying [17]. The muscle arises from a circular area below the incisors, and its fibers spread to insert into the skin overlying the chin. The upper mentalis fibers lay horizontally, and lower fibers lay vertically [16]. The motor neurons of each of the two mentalis muscles originate ipsilaterally, and account for almost 10 percent of all motor neurons in each facial motor nucleus [18].

Goldsmith in 1927 described a family with hereditary geniospasm and suggested that the character of the chin movement in offspring appeared to be intensified by procreating with “high tempered mates” [19]. Since that time, little progress has been made to elucidate the precise genetic basis of the disorder. Most cases described in the literature have been hereditary with an autosomal dominant inheritance pattern and high penetrance [13]. There have been two sporadic cases described [4,5]. Three of our four cases were sporadic, suggesting that sporadic cases may be more common than previously appreciated. A genome-wide linkage study in 1997 suggested a causative locus of 9q13-q21 in one affected family, that did not appear causative in a second affected family [17,32]. Since these two studies utilizing linkage analysis, there have been no newer studies evaluating for causative genes using next-generation sequencing. It is possible that through utilization of more recent advances in genetic testing, we may find sequencing differences that account for the pathology of geniospasm. Other genetic etiologies that would not be captured by exome sequencing which could be causative in this disorder include trinucleotide repeats, deep intronic and regulatory element variants, or structural variants.

Electromyography (EMG) has demonstrated that each of the paired mentalis muscles contracts at the same time during geniospasm [18], with both rhythmic and arrhythmic discharges of normal motor units [10]. The origin of the movement is thought to occur from loss of inhibition or hyperexcitability of central projections to the facial nuclei [20]. It has been detected during sleep using EMG [5].

An association exists between geniospasm and recurrent nocturnal tongue-biting (RNTB), the latter of which is even less well-understood, and further studies including video polysomnography with EMG may further elaborate on underlying mechanisms. This symptom can be quite bothersome, awakening patients from sleep with painful lacerations, typically at the tip or sides of the tongue, compounded by repeated injury to the same area. Lacerations can be bloody, can lead to scarring in some, and in at least one case caused traumatic amputation of the tongue tip [8,9]. It has been described as the presenting symptom of geniospasm in several cases [5,8,9]. Biting tends to begin between 10 and 18 months and may abate or decrease during early childhood. It can occur during more than half the nights of the week and can occur more than once per night [9]. Patient three’s RNTB began at 11 months of age, at which time he began awakening multiple times per night (max 25 times) with resultant lacerations (Figure 1). It became less frequent two months later. His mother continues to have rare nocturnal tongue-biting in adulthood.

While benzodiazepines such as clonazepam generally are insufficient to treat the geniospasm itself, it appears to be helpful in treating the nocturnal biting [9]. We suggest using dosages between 0.01 and 0.1 mg/kg at bedtime in children, with gradual titration. RNTB in geniospasm has never been captured on video polysomnography, and the precise mechanism of tongue injury remains unclear. Johnson et al in 1971 reported that it appeared to be caused by hypnic jerks [8]. However, hypnic jerks in general are common and do not routinely cause such reliable tongue injury. The consistency with which patients bite their tongues during sleep suggests that the biting may be due to sleep-related faciomandibular myoclonus, a type of focal hypnic jerk that has since been described and is known to cause similar injury, and typically is responsive to treatment with clonazepam [21]. Worthy of note, patient three also is noted to regularly bite and lacerate his tongue when he sneezes.

Although for many with milder symptoms, geniospasm is an issue only of cosmesis, the movements can appear quite impressive and have been reported to cause significant social anxiety and embarrassment [20], as was the case with patient 1. There are reports of patients attempting to hide the movements by biting their lower lip or wearing a scarf [12]. Chin movements can occur of an amplitude and force sufficient to interfere with speech and drinking from a cup [9]. There have been cases described with other neurologic conditions associated such as nystagmus, strabismus, hereditary sensory motor neuropathy type 1 [16], otosclerosis [22,23], Parkinson disease [24], and REM behavior disorder [8], however the reports are limited and more likely reflect incidental associations [10].

Numerous treatments have been attempted for geniospasm. In 1930, Frey treated a family’s “quivering chins” with faradic current and ultraviolet light without much improvement. Family members also underwent a trial of psychotherapy, with some degree of improvement [25]. There is a single report of a patient who achieved remission after suffering a blow to the chin at age 13 [26]. Other treatments used have included dopamine receptor blocking agents, anticonvulsants, benzodiazepines, beta-blockers, antihistamines, and others with inconsistent results [14].

More recently, injections of botulinum neurotoxin (BoNT) have been utilized to treat geniospasm [7,26]. There are 7 major serotypes of BoNT (BoNT/A-G), each of which acts by inhibiting the release of acetylcholine from the presynaptic nerve terminal by interfering with fusion of the synaptic vesicle with the plasma membrane [27,28,30]. Botulinum toxin has been used clinically since 1977, when ophthalmologist Alan Scott first used it in the treatment of strabismus [29]. Since that time, it has been shown to be efficacious in the treatment of innumerable types of cramps, spasms, and involuntary movements, including dystonia, spasticity, and hemifacial spasm [30]. The first reported use of BoNT for geniospasm was in 1992 by Gordon, who injected a father and his two sons. Each patient received 5 units of onabotulinumtoxinA into each mentalis muscle, after which chin trembling completely resolved for periods of 2–3 months following injections. The family was followed for 5 years, during which the trembling was adequately controlled without need for increasing dosage, there were no adverse effects, and the family reported that treatment “significantly improved their quality of life” [26]. Effective doses of onabotulinumtoxinA for bothersome geniospasm range from 5 to 30 units to each mentalis muscle. The treatment appears to be beneficial and well-tolerated for those with bothersome geniospasm.

## Conclusion

Due to the rarity of the condition, limited data exist with regard to the effective treatment of geniospasm. Several interventions have been tried historically, with variable results. Our results, together with those of prior reported cases, support the use of botulinum toxin injections for the management of this condition. We recommend the use of clonazepam for recurrent nocturnal tongue-biting if present. We suggest that the tongue-biting itself may be due to an association between geniospasm and sleep-related faciomandibular myoclonus; video polysomnography with EMG will be useful to determine this.
